# Epigenetic Regulation of HIV-1 Sense and Antisense Transcription in Response to Latency-Reversing Agents

**DOI:** 10.3390/ncrna9010005

**Published:** 2023-01-10

**Authors:** Rui Li, Isabella Caico, Ziyan Xu, Mohammad Shameel Iqbal, Fabio Romerio

**Affiliations:** Department of Molecular and Comparative Pathobiology, Johns Hopkins University School of Medicine, Baltimore, MD 21205, USA

**Keywords:** HIV-1, antisense RNA, non-coding RNA, epigenetic regulation, negative-sense promoter, histone deacetylation, histone methylation, latency-reversing agents, shock and kill

## Abstract

Nucleosomes positioned on the HIV-1 5′ long terminal repeat (LTR) regulate sense transcription as well as the establishment and maintenance of latency. A negative-sense promoter (NSP) in the 3′ LTR expresses antisense transcripts with coding and non-coding activities. Previous studies identified *cis*-acting elements that modulate NSP activity. Here, we used the two chronically infected T cell lines, ACH-2 and J1.1, to investigate epigenetic regulation of NSP activity. We found that histones H3 and H4 are present on the 3′ LTR in both cell lines. Following treatment with histone deacetylase inhibitors (HDACi), the levels of H3K27Ac increased and histone occupancy declined. HDACi treatment also led to increased levels of RNA polymerase II (RNPII) at NSP, and antisense transcription was induced with similar kinetics and to a similar extent as 5′ LTR-driven sense transcription. We also detected H3K9me2 and H3K27me3 on NSP, along with the enzymes responsible for these epigenetic marks, namely G9a and EZH2, respectively. Treatment with their respective inhibitors had little or no effect on RNPII occupancy at the two LTRs, but it induced both sense and antisense transcription. Moreover, the increased expression of antisense transcripts in response to treatment with a panel of eleven latency-reversing agents closely paralleled and was often greater than the effect on sense transcripts. Thus, HIV-1 sense and antisense RNA expression are both regulated via acetylation and methylation of lysine 9 and 27 on histone H3. Since HIV-1 antisense transcripts act as non-coding RNAs promoting epigenetic silencing of the 5′ LTR, our results suggest that the limited efficacy of latency-reversing agents in the context of ‘shock and kill’ cure strategies may be due to concurrent induction of antisense transcripts thwarting their effect on sense transcription.

## 1. Introduction

The mechanisms that regulate HIV-1 gene expression have been the focus of intense research for over three decades. This process involves complex and dynamic interactions among a number of players, including *cis*-acting elements in the proviral genome, positive and negative host transcription factors, viral transactivators, nascent viral transcripts, chromatin-remodeling complexes, and variably modified histones (for a comprehensive review, see [1].

The U3 region of the 5′ long terminal repeat (LTR) contains the core promoter with a TATA box and three Sp1 binding sites, the enhancer with binding sites for NF-κB, NFAT, STAT5 and AP-1 [2,3,4,5,6], and modulatory sequences with binding sites for the host repressors YY1/LSF, CBF-1, CTIP2 and BRD2 [7,8,9,10,11,12]. The R region of the HIV-1 5′ LTR encodes the trans-activating response (TAR) stem–loop structure at the 5′ end of nascent HIV-1 transcripts that is bound by the HIV-1 Tat trans-activator [13,14]. 

During cell quiescence, HIV-1 expression is turned off and HIV-1 lies dormant. This is due to sequestration in the cytoplasm of positive transcription factors, and nuclear localization of negative transcription factors. In addition, the positive transcription elongation factor-b (P-TEFb) is absent [15], and the nucleosomes Nuc-0 and Nuc-1 are positioned on the U3 and R region, respectively, of the 5′ LTR [16]. In particular, the SWI/SNF chromatin-remodeling complex, BAF precisely positions Nuc-1 on the 5′ LTR irrespective of the proviral integration site [17]. During proviral latency, Nuc-0 and Nuc-1 display a pattern of deacetylated and methylated histone residues typical of repressed chromatin [18,19]. The histone methyltransferase, EZH2 (a subunit of the Polycomb Repressor Complex 2, PRC2) is responsible for trimethylation of lysine 27 on histone H3 (H3K27me3) [18]. PRC2 also provides a docking site for histone deacetylase 1 (HDAC1) and DNA methyltransferase 1 (DNMT1) [20,21,22]. G9a is the histone methyltransferase that deposits the H3K9me2 mark, which also plays a role in epigenetic silencing of HIV-1 [23]. Under these conditions, the RNA polymerase II (RNPII) machinery generates short transcripts [24], which are the product of premature termination caused by two host complexes: the DRB sensitivity-inducing factor (DSIF) and the negative elongation factor (NELF) [25,26]. 

Upon cell activation via cytokine- or T cell receptor (TCR)-mediated stimulation, P-TEFb is formed but remains sequestered in an inactive form in a complex with the 7SK small nuclear ribonucleoprotein and the repressor protein HEXIM1/2 [27]. Concurrently, positive transcription factors translocate into the nucleus inducing HIV-1 expression [28]. Among them, NF-κB contributes to recruit histone acetyltransferases to the 5 ‘LTR, which induces the loss of the BAF complex and its replacement with PBAF. This repositions Nuc-1 and facilitates LTR-driven transcription leading to the expression of Tat, which rescues P-TEFb from its inactive form, recruits it to the TAR element where it induces hyperphosphorylation of RNPII, leading to efficient transcription elongation.

Several studies starting in the early 1990s showed that the HIV-1 proviral genome encodes antisense transcripts [29,30]. Initial evidence from acutely and chronically infected cell lines was later extended to PBMC from early-stage, asymptomatic patients [30,31]. Indeed, antisense transcription appears to be a feature of many human and animal retroviruses [32]. Our group has independently confirmed the expression of HIV-1 antisense transcripts in multiple cell systems, including in chronically infected cell lines, acutely infected primary human CD4+ T cells, and resting CD4+ T cells isolated from peripheral blood of ART-suppressed HIV-1 [33]. To avoid the possibility of endogenous and/or self-priming [34], we developed a strand-specific RT-PCR assay [33,35]. Several other studies directly or indirectly confirmed antisense transcription within the HIV-1 genome using various models [36,37,38,39,40,41,42,43,44,45].

HIV-1 antisense transcripts are bifunctional RNAs with both coding and non-coding activities [32]. They contain an open reading frame encoding for an antisense protein (ASP) of ~190 amino acids with no known homologs and still unknown function. Our studies with chronically infected cell lines showed that during non-productive viral infection, ASP presents a sub-nuclear distribution. Following cell stimulation and during productive viral infection, ASP translocates to the cytoplasm and becomes associated with the cell surface. Moreover, after viral budding and release, ASP is present on the surface of HIV-1 virions [46]. At the same time, HIV-1 antisense transcripts act as non-coding RNAs with regulatory activity. Studies from our and other groups showed that HIV-1 antisense RNAs promote HIV-1 latency via epigenetic silencing of the 5′ LTR [33,40]. 

The HIV-1 long terminal repeats are bidirectional promoters [47], and the expression of HIV-1 antisense transcripts is dependent on a negative-sense promoter (NSP) located in the U3 region of the 3′ LTR [31]. NSP is a TATA-less and Tat-independent promoter that relies on both housekeeping and inducible transcription factors, such as Sp1, NF-κB, LEF-1, Ets-1, and USF. The start site(s) of HIV-1 antisense transcripts have been mapped near Initiator Elements (InR) located in proximity of the U3-R boundary in the 3′ LTR [37,45]. 

While the two HIV-1 LTRs share perfect sequence identity, it is still unknown whether 3′ LTR-driven antisense transcription is under the same epigenetic regulatory mechanisms as the 5′ LTR. Addressing this question is of interest in the HIV-1 cure field. The “shock and kill” approach proposes to reverse viral latency, thus exposing the infected cells to elimination through HIV-1 cytopathic effects or via immune clearance. This is achieved through an array of drugs (known as latency-reversing agents, LRAs) that induce chromatin remodeling of the 5′ LTR and increase HIV-1 transcription. Concurrent induction of antisense transcripts—which have latency-promoting activity [33,40]—could conceivably counteract the latency-reversing function of curative drugs. In the present study, we investigated the epigenetic mechanisms that regulate HIV-1 antisense transcription in the two chronically infected T cell lines, ACH-2 and J1.1. Overall, our results show that 5′ and 3′ LTRs are under similar epigenetic regulatory mechanisms, and that all LRAs we tested induce both sense and antisense transcription, which could inform the development of HIV-1 cure strategies. 

## 2. Results

### 2.1. Presence of a Nucleosome on the Proviral 3′ LTR

**Figure 1:**
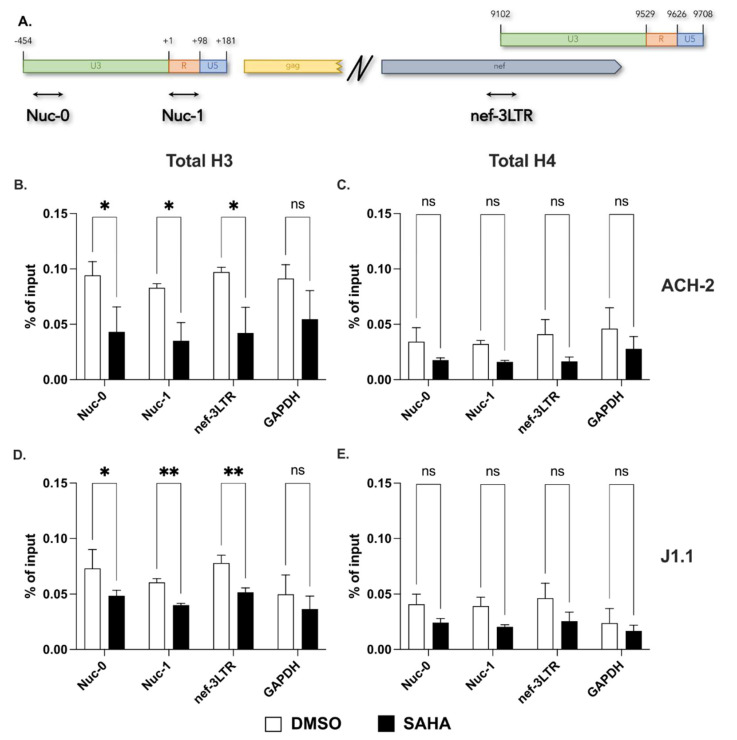
Presence of a nucleosome on the U3 region of the HIV-1 3′ LTR. (**A**) Schematic representation of the HIV-1 5′ and 3′ LTR showing the location of the three primer sets to assess the presence of histones H3 and H4 (total levels and modified residues) in ChIP assays; (**B**–**E**) Detection of histones H3 and H4 at Nuc-0, Nuc-1, nef-3LTR and GAPDH in ACH-2 (**B**,**C**) and in J1.1 (**D**,**E**) cells in DMSO− and SAHA−treated cells (open and black bars, respectively). Data show average and standard deviation (SD) of 2–4 independent experiments. To determine statistically significant differences, data were analyzed with Student’s *t*-test (unpaired, non-parametric). *, *p* < 0.05; **, *p* < 0.005; ns, not significant.

To assess whether the expression of HIV-1 antisense transcripts is under epigenetic regulation, we first performed chromatin immunoprecipitation (ChIP) assays to evaluate the presence of nucleosomes assembled on the 3′ LTR in the two non-productively, chronically infected human T cell lines, ACH-2 and J1.1. Since the two proviral LTRs share sequence identity, we identified 3′ LTR-derived sequences in qPCR by using primers mapping in the nef gene at either side of the nef—3′ LTR boundary (Figure 1A). We also performed qPCR using two primer sets that detect the previously identified nucleosomes Nuc-0 and Nuc-1 in the U3 and R region of the 5′ LTR, respectively [18]. Figure 1A shows a schematic map with the location of the three amplicons (Nuc-0, Nuc-1 and nef-3LTR). Finally, we used primers specific for the promoter region of the GAPDH gene as a control.

Cells were fixed with paraformaldehyde and then lysed. After shearing by sonication, chromatin recovered from ACH-2 and J1.1 cells was immunoprecipitated with antibodies against histones H3 and H4. Complexes were then analyzed by real-time PCR using each of the four primer sets. The data shown in Figure 1B–E show that histones H3 and H4 are present in the U3 region of the 3′ LTR at levels similar to the ones observed at Nuc-0 and Nuc-1. Following treatment with the HDAC inhibitor, SAHA we observed a decline in the levels of histones H3 and H4 at both LTRs (Figure 1B–E).

In complex, these results demonstrate that a nucleosome is positioned on the U3 region of the 3′ LTR in non-productively infected ACH-2 and J1.1 cells. Inhibition of histone deacetylation displaces the 3′ LTR nucleosome to an extent similar to that of Nuc-0 and Nuc-1 on the 5′ LTR.

### 2.2. Acetylation of Lysines on Histone H3 at the 3′ LTR

Next, we investigated histone modifications in the nucleosome present at the 3′ LTR. For these studies, we focused on two residues of histone H3 shown to be diversely modified. Modifications of lysine 9 (K9) and lysine 27 (K27) of histone H3 are often associated with either active or inactive chromatin. In particular, acetylation (Ac) in often observed in the context of active chromatin, whereas di- or trimethylation (me2 or me3) in the context of repressed chromatin [48,49,50].

**Figure 2:**
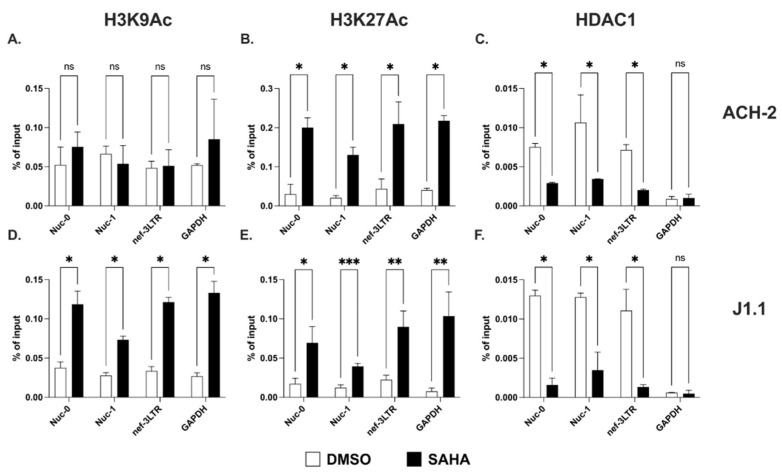
Acetylation of lysine 9 and 27 on histone H3 at the HIV-1 3′ LTR. Detection of histone H3 acetylated at lysine 9 (H3K9Ac; (**A**,**D**)) and lysine 27 (H3K27Ac; (**B**,**E**)) at Nuc-0, Nuc-1, and 3′ LTR (nef-3LTR) in untreated and SAHA-treated (open and black bars, respectively) ACH-2 and J1.1 cells (top and bottom panels, respectively). Presence of histone deacetylase 1 (HDAC1) at Nuc-0, Nuc-1, and 3′ LTR (nef-3LTR) in DMSO- and SAHA-treated (open and black bars, respectively) ACH-2 and J1.1 cells ((**C**) and (**F**), respectively). Data show average and standard deviation (SD) of 2–4 independent experiments. To determine statistically significant differences, data were analyzed with Student’s *t*-test (unpaired, non-parametric). *, *p* < 0.05; **, *p* < 0.005; ***, *p* < 0.0005; ns, not significant.

We found similar levels of acetylated lysine 9 and lysine 27 on histone H3 (H3K9Ac and H3K27Ac, respectively) at Nuc-1 and at the 3′ LTR both in ACH-2 and J1.1 cells (Figure 2A,B,D,E). Following treatment with the histone deacetylase inhibitor (HDACi), SAHA, the levels of H3K27Ac increased significantly both at Nuc-1 and the 3′ LTR in the two cell lines (Figure 2B,E). We also found increased levels of H3K27Ac at Nuc-0 (Figure 2B,E). On the contrary, SAHA treatment led to higher levels of the H3K9Ac mark at all three sites in J1.1 but not ACH-2 cells (Figure 2A,D). We also assessed the presence of the histone deacetylase 1 (HDAC1) at the three nucleosome locations. We detected the presence of HDAC1 at Nuc-0, Nuc-1 and nef-3LTR in both cell lines, and we found a significant decline following SAHA treatment (Figure 2C,F).

Thus, deacetylated H3K9 and H3K27 are found at both 5′ LTR (Nuc-0 and Nuc-1) and 3′ LTR (nef-3LTR). Treatment with the HDACi leads to a rapid increase in the levels of H3K27Ac at all three nucleosome locations in both cell lines, and to an increase in H3K9Ac levels only in J1.1 cells. Consistently with these results, the presence of HDAC1 is detectable at Nuc-0, Nuc-1 and 3′ LTR, and the levels decline following treatment with HDACi. These results suggest that histone acetylation may regulate the expression of sense and antisense transcripts with similar dynamics.

### 2.3. Methylation of Lysines on Histone H3 at the 3′ LTR

While acetylation of lysine 9 and 27 on histone H3 is an epigenetic mark indicative of decondensed and transcriptionally active chromatin, di- and trimethylation of these residues is found at transcriptionally silent chromatin. Previous studies showed the presence of H3K27me3 at the 5′ LTR of latent HIV-1 proviruses [18,33]. Thus, we sought to assess the presence and modulation of methylation at lysine 9 and lysine 27 residues of histone H3.

**Figure 3:**
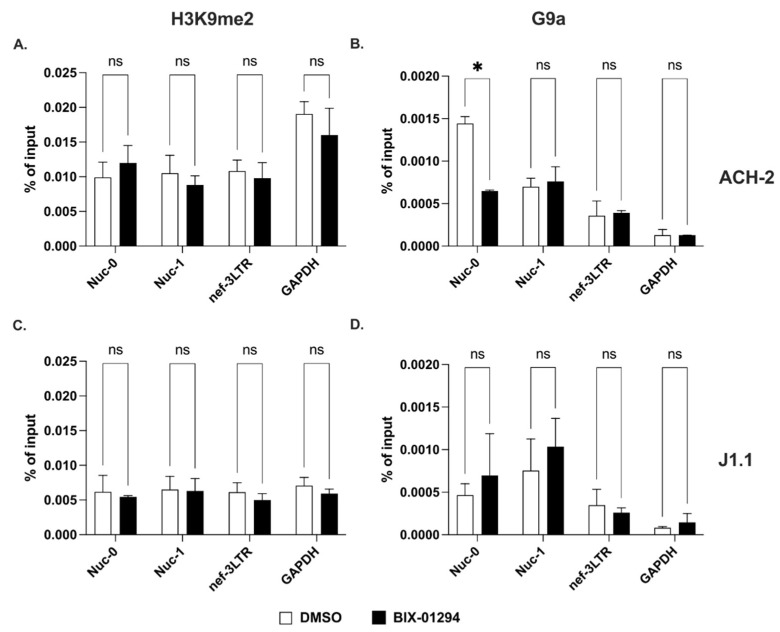
Dimethylation of lysine 9 on histone H3 at the 5′ and 3′ LTRs. (**A,C**) Detection of H3K9me2 at Nuc-0, Nuc-1, and 3′ LTR (nef-3LTR) in untreated and BIX-01294-treated (open and black bars, respectively) ACH-2 and J1.1 cells (top and bottom panels, respectively). (**B**,**D**) Presence of histone methyltransferase, G9a at Nuc-0, Nuc-1, and 3′ LTR (nef-3LTR) in DMSO- and SAHA-treated (open and black bars, respectively) ACH-2 and J1.1 cells (top and bottom panels, respectively). Data show average and standard deviation (SD) of 2–4 independent experiments. To determine statistically significant differences, data were analyzed with Student’s *t*-test (unpaired, non-parametric). *, *p* < 0.05; ns, not significant.

First, we performed ChIP assays to assess the levels of H3K9me2 at Nuc-0, Nuc-1 and 3′ LTR before in ACH-2 and J1.1 cells. We found comparable levels of this epigenetic mark at all three sites (Figure 3A,C). Interestingly, we did not observe any change in the H3K9me2 levels following treatment with the G9a inhibitor, BIX-01294 (Figure 3A,C). Treatment with higher doses of the inhibitor or for longer times proved to be toxic. These results could be explained by the very low levels of the G9a histone methyltransferase at the three HIV-1 proviral sites (Figure 3B,D).

**Figure 4:**
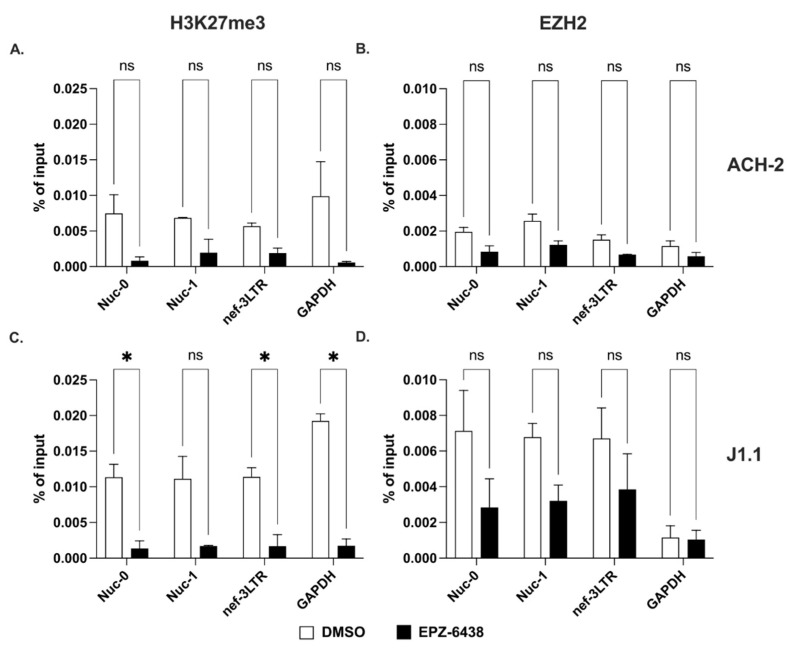
Trimethylation of lysine 27 on histone H3 at the 5′ and 3′ LTRs. (**A**,**C**) Detection of H3K27me3 at Nuc-0, Nuc-1, and 3′ LTR (nef-3LTR) in untreated and EPZ-6438-treated (open and black bars, respectively) ACH-2 and J1.1 cells (top and bottom panels, respectively). (**B**,**D**) Presence of histone methyltransferase, EZH2 at Nuc-0, Nuc-1, and 3′ LTR (nef-3LTR) in DMSO- and SAHA-treated (open and black bars, respectively) ACH-2 and J1.1 cells (top and bottom panels, respectively). Data show average and standard deviation (SD) of 2–4 independent experiments. To determine statistically significant differences, data were analyzed with Student’s *t*-test (unpaired, non-parametric). *, *p* < 0.05; ns, not significant.

Next, we measured the levels of H3K27me3 at the same three HIV-1 sites in the two cell lines. Our results showed that the suppressive epigenetic mark, H3K27me3 declined significantly at all three HIV-1 proviral sites after treatment with EPZ-6438 (Figure 4A,C). Concurrently, we observed high baseline levels of the histone methyltransferase, EZH2, which dropped significantly in response to EPZ-6438 treatment (Figure 4B,D).

Therefore, di- and trimethylation of histone H3 at positions 9 and 27 in response to specific inhibitors (BIX-01294 and EPZ-6438, respectively) behaved differently than acetylation at the same locations. Indeed, levels of H3K9me2 were substantially unchanged in response to treatment with the specific inhibitor, BIX-01294. On the contrary, the levels of H3K27me3 were profoundly affected by the specific EZH2 inhibitor, EPZ-6438.

### 2.4. Sense and Antisense Transcription in Response to HDAC and HKMT Inhibitors

**Figure 5:**
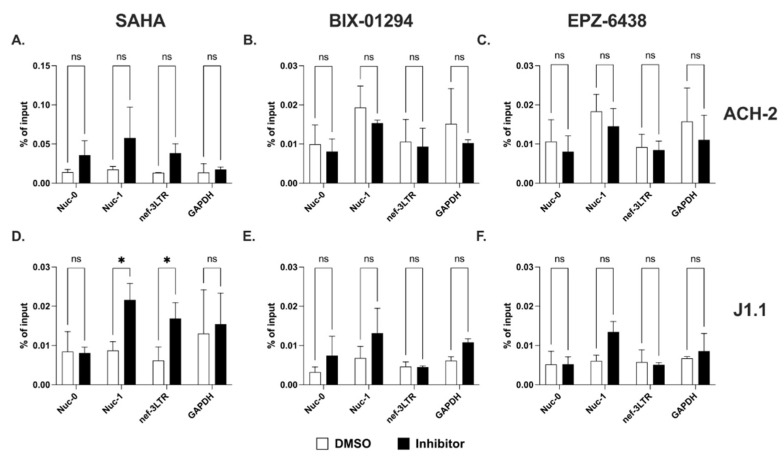
Recruitment of RNPII at Nuc-0, Nuc-1 and 3′LTR (nef-3LTR) following treatment with SAHA (**A**,**D**), BIX-01294 (**B**,**E**), and EPZ-6438 (**C**,**F**) in ACH-2 and J1.1 cells (top and bottom panels, respectively). DMSO- and inhibitor-treated samples are shown with open and black bars, respectively. Data show average and standard deviation (SD) of 2–4 independent experiments. To determine statistically significant differences, data were analyzed with Student’s *t*-test (unpaired, non-parametric). *, *p* < 0.05; ns, not significant.

We sought to investigate how histone H3 modifications affect 5′ compared to 3′ LTR-driven transcription. First, we used ChIP assays to evaluate the recruitment of RNPII at the two LTRs in ACH-2 and J1.1 cells before and after treatment with HDACi (SAHA) and the histone methyltransferase inhibitors (HKMTi; EPZ-6438 and BIX-01294). We found that SAHA treatment induced a rapid accumulation of RNPII at Nuc-1 and at the 3′ LTR in both cell lines (Figure 5A,D). However, RNPII was recruited to the Nuc-0 region in ACH-2 but not in J1.1 cells. This could represent RNPII recruited at the negative-sense promoter in the U3 region of the 5′ LTR. The two HKMTi, BIX-01294 and EPZ-6438 slightly increased accumulation of RNPII at Nuc-1 in J1.1 cell. However, they did not have any significant impact on RNPII recruitment at any other site in either cell line (Figure 5B,C,E,F).

**Figure 6:**
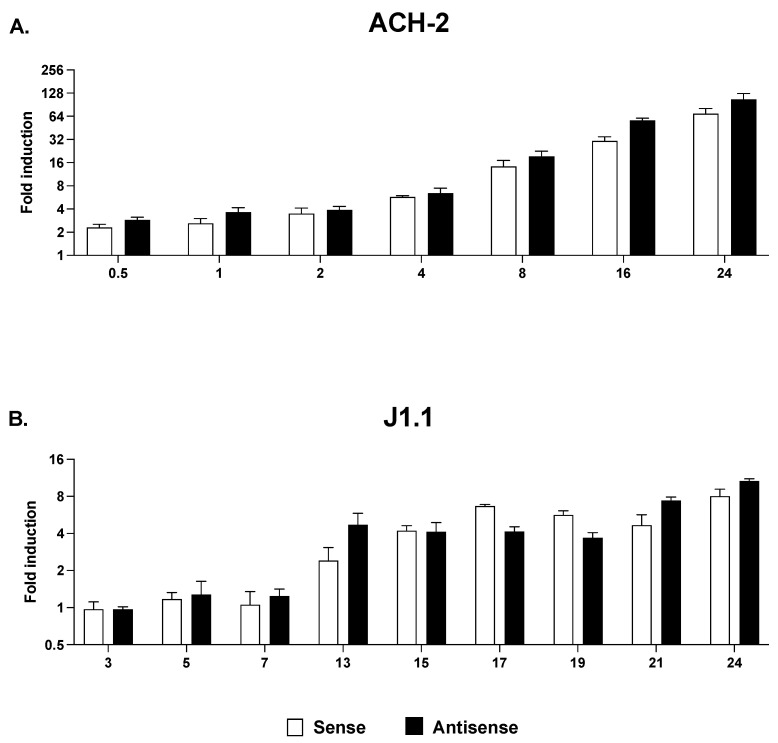
Time course of 5′ LTR-driven (open bars) and 3′ LTR-driven (black bars) transcription in ACH-2 (**A**) and J1.1 cells (**B**) following treatment with HDACi. Cells were treated with SAHA and sampled at multiple time points over 24 h. Sense and antisense RNA levels were measured by strand-specific RT-qPCR and expressed as fold induction over the levels at 0 h post-stimulation. Data show average and standard deviation (SD) of 2–4 independent experiments.

The presence of RNPII at the 3′ LTR following a short 3 h stimulation with SAHA is unlikely to reflect transcription machinery that originated at the 5′ LTR and that covered the entire genome. Indeed, we did not observe RNPII at the 3′ LTR following treatment of J1.1 cells with the two histone methyltransferase inhibitors despite the fact that both increased RNPII recruitment at Nuc-1 (Figure 5E,F). To confirm this conclusion, we sought to parallel these results with an analysis of strand-specific sense and antisense transcription in response to treatment with HDACi in the two cell lines. Cells were exposed to SAHA and sampled at various time points up to 24 h. We found that SAHA treatment induced sense and antisense with similar kinetics and to similar extent at each time point analyzed (Figure 6). Since neither BIX-01294 or EPZ-6438 affected RNPII levels at the 3′LTR, their effect on sense and antisense transcription was not evaluated.

Altogether, these results show that the HDACi, SAHA increased accumulation of RNPII at 5′ LTR and 3′ LTR promoters and led to comparable induction of sense and antisense transcripts. On the contrary, the HKMTi, BIX-01294 and EPZ-6438 caused RNPII accumulation only at the 5′ LTR and no effect was observed at the 3′ LTR. Therefore, sense and antisense transcription are under partially overlapping but not identical control mechanisms.

### 2.5. Effect of Multiple LRAs on Sense and Antisense Transcription

Our published studies showed that the HIV-1 antisense transcript *Ast* promotes viral latency through a mechanism that involves epigenetic silencing of the 5′ LTR [33]. This is consistent with previous reports showing that knocking down the expression of antisense transcripts leads to higher expression levels of sense HIV-1 transcripts [37,40]. At the same time, the results presented above indicate that SAHA—a histone deacetylase inhibitor used in “kick and kill” HIV-1 cure strategies—rapidly induces the expression of antisense transcripts. This suggests the possibility that concurrent induction of *Ast* expression following SAHA treatment may limit its efficacy in reversing latency. Therefore, we sought to identify LRAs that upregulate sense but not antisense transcription.

**Figure 7:**
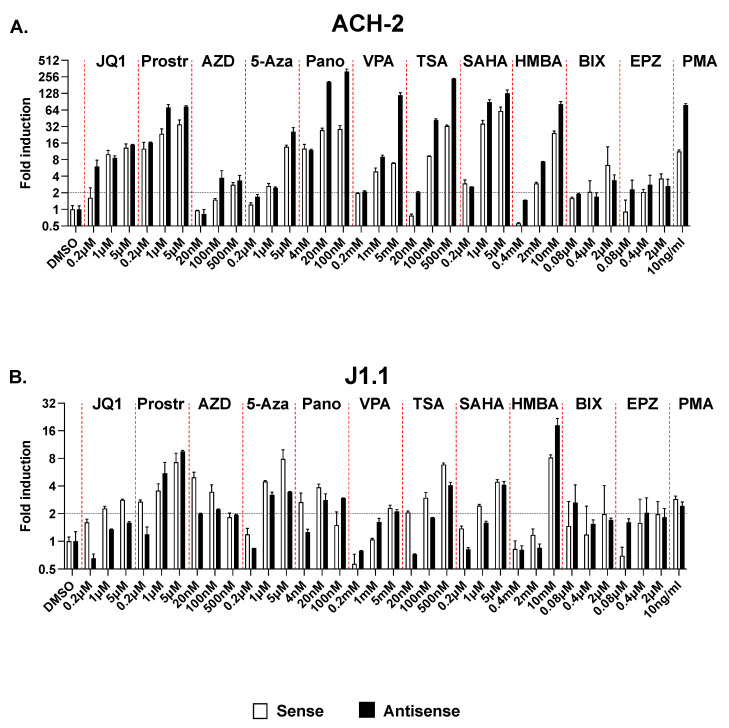
Induction of sense and antisense HIV-1 transcription in ACH-2 (**A**) and J1.1 cells (**B**) following treatment with multiple LRAs. Cells were treated with 11 different compounds at three different concentrations for 24 h (except for BIX-01294 and EPZ-6438, which were used for 3 days). As positive controls we used PMA. Sense and antisense RNA levels were measured by strand-specific RT-qPCR and expressed as fold induction over DMSO-treated controls. Data show average and standard deviation (SD) of 2–4 independent experiments. The black horizontal dotted line indicates the 2-fold induction threshold over mock-treated samples. The red vertical dotted lines separate treatment with increasing doses of each LRA. Prostr, prostratin; AZD, AZD5582; 5-Aza, 5-Azacytidine; Pano, Panobinostat; VPA, valproic acid; TSA, trichostatin A; BIX, BIX-01294; EPZ, EPZ-6438.

For these studies, we tested 11 different drugs belonging to 7 different classes of LRAs: the HDAC inhibitors Panobinostat, Valproic Acid (VPA), Trichostatin A (TCA), and SAHA; the BET inhibitor, JQ1; the canonical NF-κB pathway agonist, Prostratin; the non-canonical NF-κB pathway agonist or SMAC mimetic, AZD5582; the P-TEFb agonist, HMBA; the DNA methyltransferase inhibitor, 5-azacytidine; and the HKMTi, BIX-01294 and EPZ-6438. Each compound was tested at three different concentrations. ACH-2 and J1.1 were cultured with these compounds for 24 h (except for the two HKMTi, which were tested over 3 days). As negative and positive controls, we used cells treated with vehicle (DMSO) and PMA, respectively. At the end of the treatment, cells were harvested, and induction of sense and antisense transcripts in response to treatment with the various compounds was assessed by RT-qPCR. We found that in the ACH-2 model, all compounds induced antisense transcription to an extent similar or often greater than sense transcription (Figure 7A). In particular, at the highest concentration tested, all four HDAC inhibitors induced antisense transcription to levels significantly higher than sense transcription. In the J1.1 model, several LRAs increased sense transcription more efficiently than antisense transcription (Figure 7B). However, none of the 11 compounds tested here were able to increase sense but not antisense transcription in either cell system.

Overall, these results indicate that several classes of LRAs being investigated in the context of “kick and kill” HIV-1 cure strategies do not show a specific effect on 5′ LTR- vs. 3′ LTR-driven transcription. Since HIV-1 antisense transcripts have the ability to function as non-coding RNAs that suppress the expression of their cognate sense transcript via epigenetic silencing, they suggest the possibility that the limited efficacy of these compounds in reversing latency may be due in part to concurrent induction of antisense HIV-1 transcripts which counteract their activity at the proviral 5′ LTR.

## 3. Discussion

The HIV-1 5′ LTR drives the expression of the viral genes encoded in the positive strand of the proviral genome [1]. The *cis* elements that regulate HIV-1 transcription include the core promoter (TATA box and Sp1 binding sites), the enhancer (NF-κB, CBF-1, LEF-1, and Ets-1 binding sites), and the modulatory region (USF, NFAT, and c-Myb binding sites) [51]. The chromatin state of the 5′ LTR contributes to regulate HIV-1 expression. Two nucleosomes—termed Nuc-0 and Nuc-1—are invariably and precisely positioned on the 5′ LTR [1,51]. Formation of open and closed chromatin around these nucleosomes is regulated via histone acetylation and methylation, respectively. In particular, acetylation or methylation of lysine 9 and 27 on histone H3 at Nuc-1 represent epigenetic marks of transcriptionally active and inactive chromatin, respectively [8,18,23,52]. Indeed, HDAC inhibitors increase histone acetylation levels at the 5′ LTR and potently reactivate the expression of latent HIV-1 in multiple in vitro cell models. On the other hand, compounds that specifically inhibit different HKMT have shown different efficacies in reversing latency in vitro. Indeed, cell line models only respond to treatment with EZH2 inhibitors [18,23], whereas primary cell models also respond to treatment with G9a inhibitors [52].

The HIV-1 LTRs (as well as the those of other retroviruses) have been shown to function as bidirectional promoters [47]. Indeed, the U3 region of the 3′ LTR contains a negative-sense promoter (NSP) that drives the expression of antisense transcripts with both coding and non-coding activities [33,46]. The NSP lacks a canonical TATA box and instead relies on one or possibly multiple initiators [31,45,53]. In addition, the *cis* elements that contribute to regulate the activity of the NSP have been precisely identified [45]. 

This study provides the first evidence that the expression of HIV-1 antisense transcripts is also regulated through epigenetic mechanisms. We performed ChIP assays with antibodies directed against total and modified histones H3 and H4, and we used a primer pair that specifically detects histones present at the HIV-1 3′ LTR (Figure 1A). Our results demonstrate the presence of a nucleosome near the 5′ end of the U3 region in the 3′ LTR. We found that histones H3 and H4 are present at roughly equal molar ratios at Nuc-1 and at the 3′ LTR. Moreover, our studies show that epigenetic modifications of histones H3 and H4 at the 3′ LTR contribute to regulate the expression of antisense transcripts. Indeed, cell treatment with HDAC inhibitors led to a significant decline in the levels of H3 and H4 at the 3′ LTR. Moreover, treatment with SAHA increased the presence of histone H3 acetylated at both lysine 9 and 27, and concurrently reduced the presence of HDAC1 at the 3′ LTR. These results paralleled the situation we observed at Nuc-0 and Nuc-1, indicating that histone acetylation regulates sense and antisense transcription in similar fashion. Indeed, inhibition of histone deacetylation promoted recruitment of RNA polymerase II at the 3′ LTR and increased antisense transcription with kinetics that closely mirrored those observed for sense transcripts.

The picture that emerges from our studies of histone methylation at the 3′ LTR is less clear and, in some respects, is different than what we observed at the 5′ LTR. A 72 h treatment with the G9a-specific inhibitor, BIX-01294 had very little effect on the levels of H3K9me2 at either LTR. At the same time, BIX-01294 did not alter the recruitment of G9a at the 5′ or 3′ LTR, with the exception of Nuc-0 in ACH-2 cells. These results are consistent with the evidence that treatment with BIX-01294 did not increase the levels of RNPII at the 5′ or 3′ LTR: we observed only modest increase in RNPII levels at Nuc-1 in J1.1 cells. Indeed, following treatment with BIX-01294 we observed a modest effect on the expression of sense and antisense transcripts in both ACH-2 and J1.1, which is in line with what has been reported previously in a different HIV-1 latency model [18]. The perceived discrepancy between the effect of BIX-01294 on the levels of H3K9me2 and RNPII at Nuc-1 and at the 3′ LTR compared to its effects on sense and antisense transcription levels could be explained by the possibility that BIX-01294 treatment causes an early and transiently reduction in H3K9me2 levels and concurrent increase in RNPII levels at the two promoters, which turns on transcription of sense and antisense RNAs. After 72 h of treatment, the levels of H3K9me2 and RNPII levels return to baseline, but sense and antisense transcripts accumulated over the previous 72 h are still measurable. 

On the other hand, the EZH2-specific inhibitor, EPZ-6438 significantly reduced the levels of H3K27me3 at both LTRs. Concurrently, treatment with EPZ-6438 impacted the recruitment of EZH2 to both LTRs. This is consistent with previous evidence showing that the H3K27me3 mark is self-maintaining. Indeed, deposition of this epigenetic mark facilitates further recruitment of PRC2 (the chromatin-remodeling complex comprising EZH2), which maintains deposition of the epigenetic mark [54,55]. Thus, EPZ-6438 inhibits the enzymatic activity of EZH2 and decreases the levels of H3K27me3, which in turn leads to reduced recruitment of EZH2 at the two promoters. Once again, we observed a possible discrepancy between the impact of EPZ-6438 on recruitment of RNPII at Nuc-1 and at the 3′ LTR compared to its effect on sense and antisense transcription. Indeed, ChIP analyses performed 72 h post-treatment did not show a significant impact on the levels of RNPII at the two promoter regions (except for Nuc-1 in J1.1 cells). Nevertheless, we observed a 2–4-fold increase in the levels of both sense and antisense transcripts. Again, this could be due to the sampling time: the increased recruitment of RNPII may occur at earlier time points than the observed increase in the accumulation of sense and antisense RNAs. It should also be noted that the effect of the two HKMTi on sense and antisense transcription is relatively small compared to other compounds we tested, especially in ACH-2 cells.

The two cell lines we utilized for these studies (ACH-2 and J1.1) are infected with replication-competent HIV-1 [56,57,58] and display a varied pattern of HIV-1 integration sites and proviral orientation relative to the surrounding host gene [59]. Previous studies showed that in vivo HIV-1 integrates primarily in introns of actively transcribed genes and in both orientations relative to the sense of transcription of the host gene [60,61,62,63]. Our current knowledge also indicates that the relative sense of HIV-1 and host gene transcription does not impact the chromatin state of the HIV-1 provirus or its expression, and that the presence and positioning of nucleosomes on the provirus is independent of its integration site and the relative orientation of HIV-1 and host gene [1]. Indeed, a recent study showed that nucleosomes assemble on the HIV-1 DNA prior to integration into the host genome [64]. Moreover, the precise location of nucleosomes on the pre-integrated HIV-1 DNA closely matches that of nucleosomes assembled on the proviral DNA. With the exception of integration into constitutive heterochromatin, the consistent and precise nucleosome positioning on the proviral genome irrespective of integration site and orientation strongly suggest that the chromatin landscape in the neighboring gene does not impact the transcriptional activity of the 5′ LTR. This is in line with the evidence that HIV-1 infected cells can proliferate without inducing the expression of integrated proviruses [65]. While direct demonstration is not yet available, it is reasonable to hypothesize that the same applies to the NSP in the 3′ LTR and antisense transcription. 

We and others have shown that HIV-1 antisense transcripts function as non-coding RNAs and suppress the expression of sense viral transcripts via epigenetic mechanisms [33,40]. In particular, our group showed that a 2.6 kb HIV-1 antisense transcript promotes viral latency via recruitment of PRC2 to the 5′ LTR and deposition of H3K27me3 [33]. Indeed, the over-expression of this antisense transcript through lentiviral transduction led to consistently high levels of H3K27me3 and EZH2 levels at Nuc-1 and concurrent suppression of sense transcription even after treatment with various LRAs [33]. This suggests the possibility that the increased expression of antisense transcripts in response to LRAs that we report in this study may thwart the effect of these compounds on sense transcription. Indeed, Saayman and colleagues reported that knocking down the expression of antisense transcripts increases the rate of sense transcription [40]. This has implications for HIV-1 cure strategies that seek to reverse latency and expose productively infected to elimination by viral cytopathic effects or immune responses, commonly known as “kick and kill”. Our studies indicate that several classes of latency-reversing agents being investigated are capable of increasing the expression of antisense transcripts, which is expected to dampen the activation of sense transcripts. Therefore, the search for new latency-reversing agents with potential clinical application should evaluate their impact on antisense transcription. Our ongoing studies show that the functional activity of the 2.6 kb HIV-1 antisense transcript involves direct interaction with the U3 region of the 5′ LTR via sequence homology. While we do not have evidence of interaction between the antisense transcript and the same region of the 3′ LTR, we cannot exclude that it may occur in vivo. Nevertheless, our studies show that the antisense transcript contributes to the assembly of Nuc-1 on the R region of the 5′ LTR, and that it suppresses positive-sense transcription from the transcription start site at the junction between the U3 and the R regions. If the antisense transcript has a similar effect at the 3′ LTR, this is not expected to impact the activity of the NSP that directs transcription in the opposite sense.

An important and often overlooked caveat of the points discussed above is the fact that they all emerge from the study of HIV-1 infection in T lymphocytes. Much less is known of the epigenetic mechanisms that regulate HIV-1 transcription in myeloid cells, such as macrophages, microglia, and dendritic cells. A recent study by Lu and colleagues examined the epigenetic marks throughout the HIV-1 proviral genome in monocyte-derived macrophages compared to primary CD4+ T cells and the latently infected Jurkat-derived T cell line, JLat [66]. This study found significant differences between epigenetic marks at the 5′ and 3′ LTRs in myeloid vs. lymphoid cells. At the same time, the epigenetic marks present at the two LTRs in infected myeloid cells did not parallel the ones observed in JLat cells, a *bona fide* latency model [66]. Moreover, the authors also detected the high expression of *Tat* and *Nef* transcripts, suggesting that myeloid cells analyzed in that study were not latently infected. Therefore, the epigenetic marks found at the two HIV-1 LTRs in non-productively infected myeloid cells still remain to be ascertained, and the possibility remains that different agents should be utilized for latency reversal in different cellular and anatomical reservoirs.

In conclusion, this study provides the first analysis of the epigenetic mechanisms that regulate 3′ LTR-driven antisense transcription in two T cell line models of HIV-1 latency. Our results demonstrate that a nucleosome is present at the HIV-1 3′ LTR. We also show that histone H3 subunits in this nucleosome are modified with suppressive epigenetic marks in a fashion similar as the ones observes at the 5′ LTR. Finally, we provide evidence that multiple agents that increase the expression of sense transcripts have similar effects on antisense transcription, which can inform future “kick and kill” cure strategies. 

## 4. Materials and Methods

### 4.1. Cell Lines and Reagents 

ACH-2 and J1.1 cells were obtained from the NIH HIV-1 Reagent Program (Germantown, MD, USA) and maintained in RPMI 1640 supplemented with 10% fetal calf serum, penicillin, streptomycin and L-glutamine (all from ThermoFisher Scientific, Waltham, MA USA) at 37 °C and 5% CO_2_. Phorbol 12-myristate 13-acetate (PMA), Prostratin, Valproic Acid (VPA), Trichostatin A (TSA), JQ1, and Suberoylanilide hydroxamic acid (SAHA) were purchased from Sigma-Aldrich (St. Louis, MO, USA). Panobinostat was from BioVision (Milpitas, CA, USA). EPZ-6438 and AZD5582 were obtained from Selleck Chem (Houston, TX, USA). BIX-01294 and 5-azacytidine were from StemCell Technologies (Vancouver, BC, Canada). Hexamethylene bisacetamide (HMBA) was obtained from Abcam (Boston, MA, USA). All inhibitors were solubilized in DMSO, except for HMBA, which was resuspended in sterile water. 

### 4.2. Reverse Transcription and Real-Time PCR (RT-qPCR) Assay

Total RNA was isolated from ~1 × 10^6^ cells using the RNeasy Mini Kit (Qiagen, Germantown, MD, USA) and 1 μg of RNA was used for reverse transcription using the iScript Select cDNA Synthesis Kit (BioRad, Hercules, CA, USA). Real-time quantitative RT-PCR (RT-qPCR) to assess antisense transcription was performed as previously described [33]. For detection of sense transcripts by RT-qPCR, we performed the RT reaction with random primers, and the qPCR with the following primers and probe: 5′-CGCCCGAACAGGGACTT-3′, 5′-CCTGCGTCGAGAGATCTCCT-3′, and 5′-CTGGCTTTACTTTCGCTTTC-3′.

### 4.3. ChIP Analysis

ChIP was performed using the SimpleChIP Plus Sonication Chromatin IP kit (Cell Signaling Technology, Danvers, MA, USA) following the manufacturer’s protocol. Briefly, cells were either treated with 5 μM SAHA, 2 μM EPZ-6438, 2 μM BIX-01294, or DMSO for the indicated times, fixed with 1% formaldehyde for 10 min at room temperature, and then quenched for 5 min by adding glycine to 125 mM final concentration. After cold PBS wash, cells were lysed, and the chromatin was sheared using the QSonica Q800R3 system for 22 min at 70% amplitude with 15s ON/45s OFF cycles. Immunoprecipitations were performed by overnight incubation of 25 μg sonicated chromatin with 4 μg of respective antibodies. PCR was performed using specific primers (Table S1). The percentage-of-input method was used to calculate the enrichment of proteins in specific regions of the HIV-1 proviral genome. The following antibodies were used for ChIP: anti-H3 (Cell Signaling Technologies, 4620), anti-H4 (Cell Signaling Technologies, 14149), anti-EZH2 (Abcam, ab191250), anti-G9a (Cell Signaling Technologies, 68851), anti-H3K9ac (Cell Signaling Technologies, 9649), anti-H3K27ac (Cell Signaling Technologies, 8173), anti-H3K27me3 (Abcam, ab6002), anti-H3K9me2 (Abcam, cat. ab1220), anti-HDAC1 (Cell Signaling Technologies, 34589), anti-RNA polymerase II (Millipore, Burlington, MA, USA, cat. 05-623), pre-immune IgG (Cell Signaling Technologies, cat. 2729).

### 4.4. Latency Reversal

For reactivation of latent HIV-1, 1 × 10^6^ cells were plated in 24-well plates and incubated with latency-reversing agents at the indicated concentrations for 24 h, except for EPZ-6438 and BIX-01294 (3 days). After LRA treatment, cells of each group were harvested for the quantification of HIV-1 sense and antisense RNA transcripts.

## Data Availability

Not applicable.

## Supplementary Materials

The following supporting information can be downloaded at: https://www.mdpi.com/article/10.3390/ncrna9010005/s1, Table S1: Sequence of primers for ChIP assays.
